# Activation of the prostaglandin D2 metabolic pathway in Crohn’s disease: involvement of the enteric nervous system

**DOI:** 10.1186/s12876-015-0338-7

**Published:** 2015-09-04

**Authors:** Anne-Gaelle Le Loupp, Kalyane Bach-Ngohou, Arnaud Bourreille, Hélène Boudin, Malvyne Rolli-Derkinderen, Marc G. Denis, Michel Neunlist, Damien Masson

**Affiliations:** 1 INSERM Unité 913, 1 rue Gaston Veil, Nantes, F-44035 France; 2 Université Nantes, 1 quai de Tourville, BP 13522, Nantes, F-44035 France; 3 Institut des Maladies de l’Appareil Digestif, 1 place Alexis Ricordeau, Nantes, F-44093 France; 4 Laboratoire de Biochimie, Institut de Biologie, CHU de Nantes, 1 place Alexis Ricordeau, Nantes, F-44093 France

## Abstract

**Background:**

Recent works provide evidence of the importance of the prostaglandin D2 (PGD2) metabolic pathway in inflammatory bowel diseases. We investigated the expression of PGD2 metabolic pathway actors in Crohn’s disease (CD) and the ability of the enteric nervous system (ENS) to produce PGD2 in inflammatory conditions.

**Methods:**

Expression of key actors involved in the PGD2 metabolic pathway and its receptors was analyzed using quantitative reverse transcriptase polymerase chain reaction (qRT-PCR) in colonic mucosal biopsies of patients from three groups: controls, quiescent and active CD patients. To determine the ability of the ENS to secrete PGD2 in proinflammatory conditions, Lipocalin-type prostaglandin D synthase (L-PGDS) expression by neurons and glial cells was analyzed by immunostaining. PGD2 levels were determined in a medium of primary culture of ENS and neuro-glial coculture model treated by lipopolysaccharide (LPS).

**Results:**

In patients with active CD, inflamed colonic mucosa showed significantly higher COX2 and L-PGDS mRNA expression, and significantly higher PGD2 levels than healthy colonic mucosa. On the contrary, peroxysome proliferator-activated receptor Gamma (PPARG) expression was reduced in inflamed colonic mucosa of CD patients with active disease. Immunostaining showed that L-PGDS was expressed in the neurons of human myenteric and submucosal plexi. A rat ENS primary culture model confirmed this expression. PGD2 levels were significantly increased on primary culture of ENS treated with LPS. This production was abolished by AT-56, a specific competitive L-PGDS inhibitor. The neuro-glial coculture model revealed that each component of the ENS, ECG and neurons, could contribute to PGD2 production.

**Conclusions:**

Our results highlight the activation of the PGD2 metabolic pathway in Crohn’s disease. This study supports the hypothesis that in Crohn’s disease, enteric neurons and glial cells form a functional unit reacting to inflammation by producing PGD2.

## Background

Inflammatory bowel diseases (IBD) are described as multifactorial pathologies with an uncontrolled immune response leading to inflammation in genetically predisposed individuals. IBD etiologies remain unresolved [1, 2]. The role of prostaglandins (PG) in its pathogenesis was first suggested in 1977 [3]. Previous studies have reported high levels of PGD2 in animal models of colitis [4, 5] and in colonic mucosal biopsies of patients with a history of ulcerative colitis (UC) [6]. PGD2 synthesis is derived from prostaglandin H2 (PGH2) through prostaglandin D synthase (PGDS). PGH2 is derived from arachidonic acid by cyclooxygenases (COX), which exist in two isoforms: COX1 and COX2. COX1 is expressed constitutively in most cell types, whereas COX2 is induced by inflammatory stimuli such as bacterial endotoxin and proinflammatory cytokines. Two distinct PGDS have also been identified: hematopoietic PGDS (HPGDS) and lipocalin-type PGDS (L-PGDS). Similarly to COX2, L-PGDS but not HPGDS is known to be inducible in inflammatory conditions [7, 8]. While overexpression of microsomal prostaglandin E synthase-1 and the proinflammatory role of PGE2 have been well described in IBD [9], the role of PGD2 in IBD remains debated and there have been no reports on PGD2 in Crohn’s Disease (CD). Ajuebor *et al*. demonstrated that PGD2 reduced granulocyte infiltration during experimental colitis [5]. Vong *et al.* reported high levels of PGD2 in the colonic tissue of patients with quiescent CD [6]. Some of the anti-inflammatory properties attributed to PGD2 may be related to the effects of its metabolite, 15-deoxy-Δ^12,14^-prostaglandin J2 (15dPGJ2), which has been shown to exert potent anti-inflammatory effects in animal models through activation of its receptor PPARG [10, 11]. Conversely, Hokari *et al*. suggested that L-PGDS plays a proinflammatory role in the development of colitis in clinical and experimental studies [12]. They reported that the level of L-PGDS mRNA expression was increased in UC patients in parallel with disease activity. However, changes in PGD2 production or in the expression of enzymes involved in its synthesis during CD are currently unknown.

Although immune cells are central producers of cytokines and prostaglandins; the cells of the mucosa are also known to be able to contribute to this production [13]. The ENS is a major constituent of the intestinal mucosal microenvironment. The ENS, composed of enteric neurons and enteric glial cells (EGCs), together with the intestinal epithelial barrier (IEB) is a functional entity, the digestive neuronal-glial-epithelial unit, based on the physical proximity and paracrin relationship between its components [14]. Indeed, the ENS is known to regulate the gut homeostasis process and, in particular, intestinal epithelial barrier functions, *via* the release of various mediators [14]. Recent studies have shown that the ENS is able to sense inflammatory stressors and respond by secreting various cytokines or chemokines [15, 16]. Furthermore, various studies have described abnormalities of the ENS in CD and have suggested a role of the ENS in the pathogenesis of IBD [17–22]. Recently, we have demonstrated that the EGCs of the ENS are involved in controlling intestinal epithelial functions through secretion of 15dPGJ2 [23]. However, the ability of the ENS to produce PGD2 and its modulation by inflammation remains unknown.

Based on these findings, our aims were to investigate expression of key actors of the PGD2 metabolic pathway in colonic mucosal biopsies of patients with CD and to evaluate the ability of the ENS to produce PGD2 in proinflammatory conditions.

## Methods

### Patient selection

Tissue samples were collected from colonic biopsies of patients with CD. Thirty patients with CD treated at the Department of Gastroenterology (Nantes University Hospital, France) were included in this study. Colonic biopsies were obtained and stored in the bio-collection of the “Institut des Maladies de l’Appareil Digestif (IMAD)”. The clinical and demographic data were collected and recorded in a computerized database securely coupled to the biological collection. This bio-collection was approved and registered by the French Ministry of Science and Research “DC-2008-402”. CD was diagnosed on the basis of clinical and endoscopic criteria. Fifteen quiescent disease patients and fifteen active disease patients, with acute or chronic inflammation, were included in this study. Two biopsies were collected from patients with active disease, one in an inflamed zone and one in a normal zone. Colonic biopsy specimens from normal mucosa were collected from fifteen patients with colonic polyps and were used as controls. All tissues were processed according to the French Guidelines for Research on Human Tissues (Agence Nationale d’Accréditation 2001). The characteristics of patients included are given in Table 1. After collection, biopsies were immediately immersed in RA1 buffer (Macherey Nagel, Hoerdt, France) and stored at – 80 °C before real-time PCR analysis. Samples intended for PGD2 determination were stored at −80 °C until time of use.

**Table 1:** Clinical characteristics and treatments - Crohn’s disease patients at the time of study inclusion. Values are given as the mean (minimum-maximum) for continuous variables and numbers (%) for categorical variables

	Quiescent Crohn’s disease	Active Crohn’s disease	Controls
Cases	15	15	15
Age, years	36 (18–63)	37 (21–76)	55 (38–76)
Sex female/male	9/8	11/4	4/11
Age at diagnosis, years	30 (19–60)	26 (15–58)	
Duration of the disease, years	7 (1–21)	12 (1–26)	
Current treatment			
No medication	1 (7 %)	5 (33 %)	
5-ASA/Salazopyrin/Azathioprine	2 (17 %)	2 (17 %)	
Corticosteroids/immunosuppressants	9 (60 %)	6 (40 %)	
Infliximab	8 (53 %)	5 (33 %)	

For immunofluorescence staining, tissue specimens were collected from patients who underwent surgery for colonic adenocarcinoma (four colonic specimens from two patients were stained). Patients gave their informed and written consent for the biopsy studies. Specimens were taken at a distance from the tumor, in macroscopically and histologically normal areas, and immediately processed in the Pathology Department. According to the guidelines of the French Ethics Committee for Research on Human Tissues, these specimens were considered as “residual tissues”, not relevant to pathological diagnosis. Tissue samples were subsequently fixed in 4 % paraformaldehyde for 3 hours at room temperature. Following several washes in phosphate buffer saline (PBS), tissue was pinned and whole mounts of mucosal and submucosal plexus were obtained by microdissection under a microscope SZ3060 (Olympus, Rungis, France), as previously described [24].

### Cell culture and treatments

#### Primary culture of the enteric nervous system

Primary culture of the rat ENS was performed as previously described [25]. Experiments were compliant with journal policies and UK regulations on animal experimentation as described by Drummond [26]. Pregnant Sprague–Dawley rats at stage E15 were purchased (CERJ, Le Genest St Isle, France) and manipulated in compliance with French institutional guidelines. These procedures were approved by the local institutional animal research committee (Certificate E. 44011; Inserm, Nantes, France). Every effort was made to minimize animal suffering and the number of animals used. Rats were killed by an overdose of CO_2_ followed by severing of the carotid arteries. Intestinal cells obtained after dissection and dissociation were counted and then seeded at a density of 2.4 x 10^5^ cells cm^2^ on 24-well plates (Corning, Avon, France) previously coated with a 0.5 % gelatin solution (Sigma-Aldrich, Lyon, France). Primary cultures were maintained for 14 days with half of the medium replaced every day (Dulbecco’s modified Eagle medium-F12 1:1 containing 1 % N-2 supplement (Life Technologies, Cergy Pontoise, France). Primary cultures were treated for 24 h with 0.1 μg/mL LPS (Sigma-Aldrich, Lyon, France) with or without 95 μM AT-56 (provided by Y Urade; Osaka Bioscience Institute) a selective and competitive inhibitor of L-PGDS [27]. Three experiments were performed. The ENS culture media were collected, centrifuged and stored at −80 °C for further analysis. For immunofluorescence staining, primary cultures of rats’ ENS were fixed in 4 % paraformaldehyde followed by several washes in phosphate buffer saline (PBS).

#### Neuro-glial coculture model

First, a glial feeder layer was made up from the enteric glial cell line JUG2 derived from the intestine of 15-day-old rat embryos [28]. Briefly, the enteric glia were plated at a density of 7,500 cells/cm^2^ in a 24-well plate, and maintained for 4 days in DMEM containing 10 % fetal calf serum, 2 mM glutamine, 50 μg/ml streptomycin and 50 UI/ml penicillin. The medium was replaced with serum-free Neurobasal/B27 medium (Gibco) 3 h before neuron culture. Rat enteric neuron culture was prepared from 15-day-old rat embryos by previously described methods [5], except that the dissociated cells were plated at 175,000 cells/cm^2^ on glass coverslips coated with poly-L-lysine (1 mg/ml, Sigma) in DMEM high glucose containing 10 % FCS, 2 mM glutamine, 50 μg/ml streptomycin and 50 UI/ml penicillin. The coverslips were then transferred 3 h later to the wells containing the glial feeder layer and maintained for up to 7 days in Neurobasal/B27 containing 50 μg/ml streptomycin and 50 UI/ml penicillin. This procedure resulted in a culture on the coverslips composed of 82.6 ± 5.5 % of neurons and 10.6 ± 3.6 % of myofibroblasts, while no glial cells were detected (personal observation). This neuro-glial coculture system made it possible to physically separate the enteric neurons and the glial cells in order to examine the response of each cell type to LPS treatment.

### RT-PCR analysis

Total RNA was extracted from human mucosal biopsies using the Nucleospin RNA/Protein kit (Macherey-Nagel, Hoerdt, France). cDNA was synthesized using standard procedures as previously described [24]. Real-time PCR was performed according to previous reports with some modifications [29]. Primers were designed from the sequence of human cDNAs using the Universal Probe Library assay design center (https://lifescience.roche.com/webapp/wcs/stores/servlet/CategoryDisplay?tab=Assay+Design+Center&identifier=Universal+Probe+Library&langId=-1); sequences are reported in Table 2. Amplification conditions of cPLA2-alpha, COX1, COX2, IL1B, TNF-alpha, L-PGDS, HPGDS, D-type prostanoid (DP) receptor 1 and 2 (DP1, DP2), PPARG and MRPS6 templates were optimized for the Rotorgene 3000 instrument (Qiagen, Courtaboeuf, France). PCR amplifications were performed in duplicate using Light cycler 480 SYBR Green I master mix (Roche Diagnostics, Mannheim, Germany) according to the manufacturer’s protocol. External standard curves were generated with serial 5-fold dilution of cDNA samples prepared with RNA extract from human mucosal biopsies. The relative amount of transcripts was calculated from these standard curves using RotorGene software. Expression of S6 ribosomal proteins as internal controls was analyzed at the same time. For each sample, the ratio between the relative amount of each specific transcript and S6 was calculated to compensate variations in the quantity and quality of starting mRNA.

**Table 2:** Primers for RT-PCR analysis

Gene	Accession number	Species	Primer sequences (5′-3′)
TNF	NM_000594	Homo Sapiens	Fw: CGCTCTTCTGCCTGCTGCACT
			Rev: ACTGGAGCTGCCCCTCAGCTT
IL1B	NM_000576.2	Homo Sapiens	Fw: AAAGCTTGGTGATGTCTGGTC
			Rev : GGACATGGAGAACACCACTTG
PTGS1 (COX1)	NM_000962.2	Homo Sapiens	Fw: TCCTGTTGGTGGACTATGG
			Rev: GTGGTGGTCCATGTTCCTG
PTGS2 (COX2)	NM_000963.2	Homo Sapiens	Fw: TGGGAAGCCTTCTCTAACCTC
			Rev: TCAGGAAGCTGCTTTTTACCTT
PLA2G4A (cPLA2)	NM_024420.2	Homo Sapiens	Fw: TGCTACCTACGTTGCTGGTCT
			Rev: TTCTCTGGAAAATCAGGGTGA
PTGDS (L-PGDS)	NM_000954.5	Homo Sapiens	Fw: AGAAGAAGGCGGCGTTGTCC
			Rev: CCACCACTGACACGGAGTAGG
HPGDS	NM_014485.2	Homo Sapiens	Fw: GAGAATGGCTTATTGGTAACTCTGT
			Rev: AAAGACCAAAAGTGTGGTACTGC
MRPS6	NM_001010	Human mouse rat	Fw: CCAAGCTTATTCAGCGTCTTGTTACTCC
			Rev: CCCTCGAGTCCTTCATTCTCTTGGC
PTGDR (DP1)	NM_000953	Homo Sapiens	Fw: CCTGGAGGAGCGGATCA
			Rev: GCTCCATAGTAAGCGCGATAAA
PTGDR2 (DP2)	NM_004778	Homo Sapiens	Fw: CCTGTGCTCCCTCTGTGC
			Rev: TCTGGAGACGGCTCACTG
PPARG	NM_138711.3	Homo Sapiens	Fw: AAAGTCGTCCTTCCCGCTGACCAAA
			Rev: GATGGCCACCTCTTTGCTCTGC

### Immunofluorescence staining

All antibodies were diluted in PBS with 1 mg mL^−1^ sodium azide, 4 % horse serum and 1 % Triton X-100. Primary cultures of rat ENS, human plexi were incubated with PBS sodium azide horse serum and Triton X-100 followed by incubation with rabbit polyclonal anti-L-PGDS (1:500; Cayman, Spi-Bio, Montigny-le-Bretonneux, France) or with human polyclonal anti-HPGDS (1:500; Cayman Spi-Bio, Montigny-le-Bretonneux, France) overnight at 4 °C. After extensive rinsing in PBS, primary culture or human tissues were incubated with donkey anti-Rabbit IgG conjugated with carboxy-methyl-indocyanin (1:500; Beckman Coulter, Roissy, France) and donkey anti-human IgG conjugated with carboxy-methyl-indocyanin (1:500; Molecular Probes) for 3 h at room temperature. After L-PGDS and HPGDS staining, primary culture or human tissues were incubated with goat polyclonal anti-S100β (1:100; Santa-Cruz, Tebu-Bio, Le Perrayen Yveline, France) or with mouse anti-Hu (1:200 Molecular Probes) overnight at 4 °C followed by donkey anti-goat IgG conjugated with alexa Fluor 488 (1:400; Molecular Probes), donkey anti-mouse IgG conjugated with Alexa Fluor 488 fluorescent (1:500; Molecular Probes). Following washes, stained samples were observed and acquired with a IX 50 microscope (Olympus).

### PGD2 assay

PGD2 levels in colonic mucosal biopsies of CD patients or released from cell culture in incubation medium were determined using the Prostaglandin D2-MOX EIA kit (Cayman Chemical, MI, USA). For colonic mucosal biopsies, acetone extraction was performed prior to deproteinization and to concentration. Biopsies were ground in 500 μl cold acetone on ice. Precipitated proteins were removed by centrifuging at 3,000 g for 10 minutes. The sample was then evaporated to dryness under a stream of nitrogen gas. Finally, the sample was resuspended in 100 μl buffer and methoximation was performed according to the assay protocol.

### Statistical analysis

Statistical analyses were performed using Prism 4.0 (GraphPad Software Inc., La Jolla, USA). Experimental data were compared using a non-parametric Mann–Whitney test, Kruskall-Wallis test followed by Dunn’s *post hoc* test, Spearman test or Wilcoxon paired test. A *p* value < 0.05 was considered to be significant.

## Results

### mRNA expression of proinflammatory cytokines is increased in colonic biopsies from patients with active CD

**Fig. 1:**
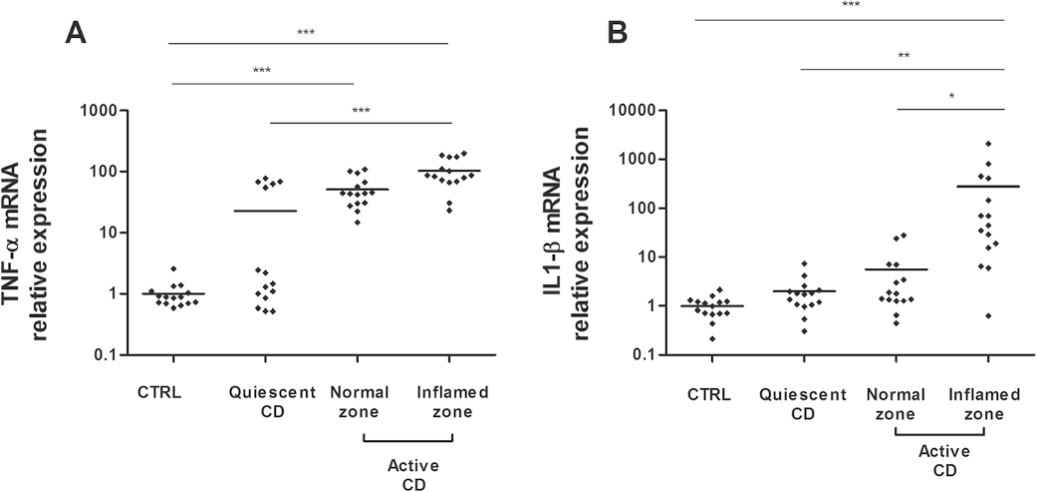
Quantitative RT-PCR analysis of TNF-alpha (**a**) and IL1B (**b**) gene expression in colonic biopsies from healthy subjects (CTRL), CD patients with quiescent disease or active disease (normal zone and inflamed zone). Data were normalized to MRPS6 gene expression (*n* = 15; ***p <0.001; **p < 0.01; *p < 0.5, Kruskall-Wallis test followed by Dunn’s *post hoc* test)

To determine the inflammatory status of the colonic biopsies from different groups of patients, we measured mRNA expression of proinflammatory cytokine tumor necrosis factor-alpha (TNF-alpha) and interleukin1,beta (IL1B). TNF-alpha mRNA was significantly increased in patients with active disease compared to healthy subjects and patients with quiescent disease (Fig 1a). Five patients with quiescent disease also presented with an increase in TNF-alpha expression. This transcriptional status was equivalent to that of patients with active CD. This increase was not associated with any relevant clinical characteristics or treatment. IL1B mRNA was significantly increased in sites of intestinal inflammation in patients with active CD compared to the other three groups: controls, biopsies from patients with quiescent disease and from normal mucosa of patients with active CD (Fig 1b).

### L-PGDS mRNA expression and PGD2 levels were increased in inflamed colonic mucosa of patients with active CD

**Fig. 2:**
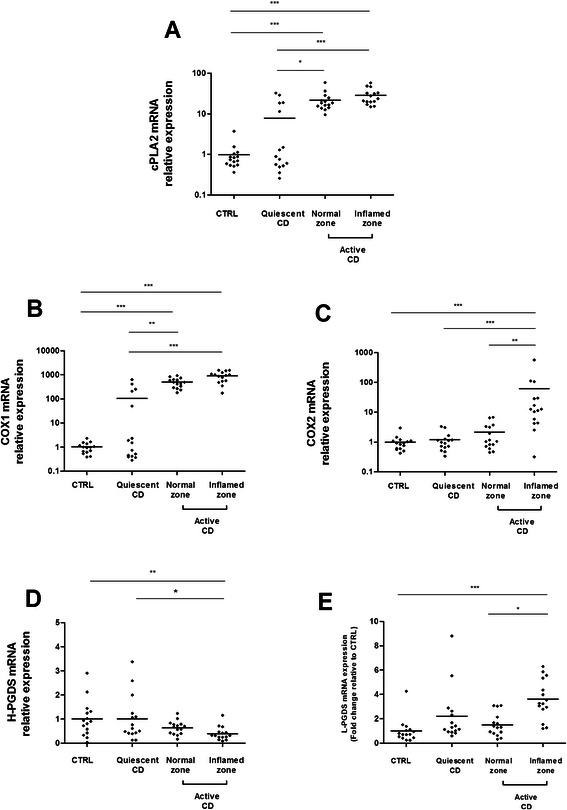
Quantitative RT-PCR analysis of cPLA2 (**a**), COX1 (**b**), HPGDS (**c**), COX2 (**d**), L-PGDS **(e**), gene expression in colonic biopsies from healthy subjects (CTRL), CD patients with quiescent disease or active disease (normal zone and inflamed zone). Data were normalized to MRPS6 gene expression (*n* = 15; ***p <0.001; **p < 0.01; *p < 0.5, Kruskall-Wallis test followed by Dunn’s *post hoc* test)

We then examined mRNA expression of the five key enzymes involved in PGD2 production and its receptors. Cytoplasmic Phospholipase A2 (cPLA2-alpha) and COX1 were up-regulated in the mucosa of patients with active CD and in the mucosa of five patients with quiescent disease (Fig 2a-b). In patients with active CD, COX2 mRNA expression was significantly increased in the inflamed zone compared to the normal zone (Fig 2c). Colonic expression of HPGDS in colonic biopsy specimens from patients with quiescent CD did not differ significantly from that of controls (Fig 2d). HPGDS mRNA levels were reduced in the inflamed zone of patients with active CD. L-PGDS mRNA expression was significantly higher in the inflamed colonic mucosa of patients with active CD than in normal mucosa of CD patients or controls (Fig 2e). TNF-alpha mRNA expression was significantly correlated with cPLA2-alpha (*r* = 0.8245; *p* < 0.0001) and COX1 (*r* = 0.84; *p* < 0.0001) expression while IL1B mRNA expression was significantly correlated with COX2 (*r* = 0.82, *p* < 0.0001) and L-PGDS (*r* = 0.48; *p* < 0.0001) expression. Only the inflamed zone in active CD showed total activation of the PGD2 metabolic pathway with up-regulation of cPLA2-alpha, COX1, COX2 and L-PGDS.

**Fig. 3:**
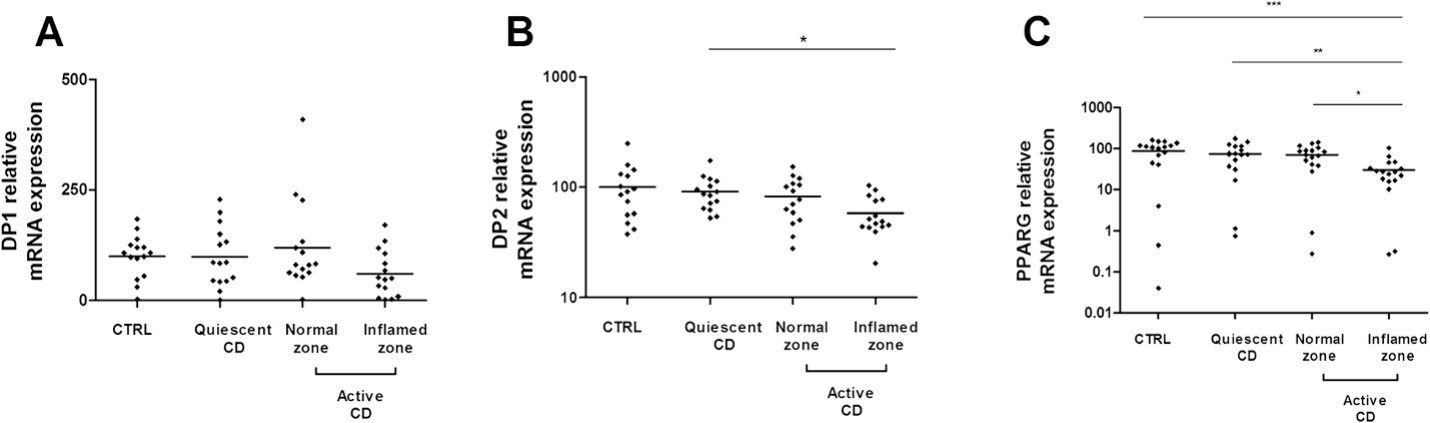
Quantitative RT-PCR analysis of DP1 (**a**), DP2 (**b**), PPARG (**c**) gene expression in colonic biopsies from healthy subjects (CTRL), CD patients with quiescent disease or active disease (normal zone and inflamed zone). Data were normalized to MRPS6 gene expression (*n* = 15; ***p <0.001; **p < 0.01; *p < 0.5, Kruskall-Wallis test followed by Dunn’s *post hoc* test)

We quantified DP1, DP2 and PPARG mRNA expression in the same samples (Fig 3). The colonic expression of DP1 and DP2 in colonic biopsy specimens from patients with CD did not differ significantly from that of controls except for the expression of DP2 which was reduced in inflamed colonic mucosa of CD patients with active disease compared with mucosa of CD patients with quiescent disease (Fig 3a-b). PPARG expression was reduced in inflamed colonic mucosa of CD patients with active disease compared with non-inflamed mucosa of CD patients with active disease, mucosa of CD patients with quiescent disease or control mucosa (Fig 3c).

**Fig. 4:**
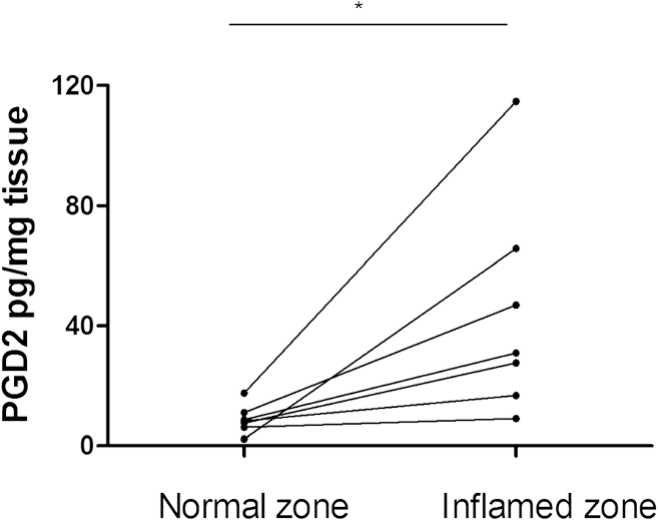
PGD2 assays in paired colonic mucosal biopsies from patients with active CD. Normal zone (*n* = 7) versus inflamed zone (*n* = 7). (*P < 0.5, Wilcoxon paired test)

To determine whether activation could lead to higher PGD2 production, we measured PGD2 levels in the colonic biopsies of CD patients. In patients with active CD, PGD2 levels were significantly higher in inflamed colonic mucosa than in normal colonic mucosa (Fig 4).

### Neurons of the enteric nervous system express Lipocalin prostaglandin D synthase

**Fig. 5:**
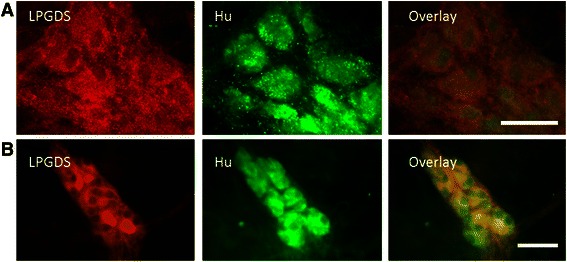
Immunofluorescence staining of L-PGDS in human plexi. In human myenteric (**a**) and submucosal plexi (**b**), Hu was colocalized with L-PGDS. Scales bars are 40 μm (**a**-**b**). Typical picture of four colonic tissues obtained in healthy area of two patients who underwent surgery for colonic adenocarcinoma

L-PGDS expression by EGCs has been previously described [23]. To determine whether enteric neurons could also express L-PGDS, the main enzyme responsible for PGD2 synthesis in inflammatory conditions, we performed L-PGDS immunofluorescence labeling in human myenteric (Fig 5a) and in human submucosal plexi (Fig 5b). Neurons were selectively stained using antibodies directed against the neuronal protein Hu C/D. We found that L-PGDS was expressed in neurons of both human myenteric and submucosal plexi. HPGDS was not expressed by ENS components (data not provided).

### ENS produced PGD2 *via* L-PGDS in proinflammatory conditions

**Fig. 6:**
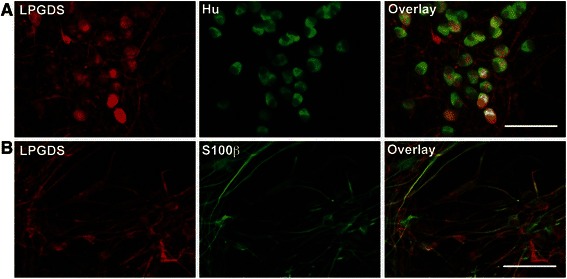
Immunofluorescence staining of L-PGDS in primary culture of rat ENS. In primary culture of rat ENS, Hu (**a**) and S100β (**b**) were colocalized with L-PGDS. Scales bars are 50 μm (A-B)

**Fig. 7:**
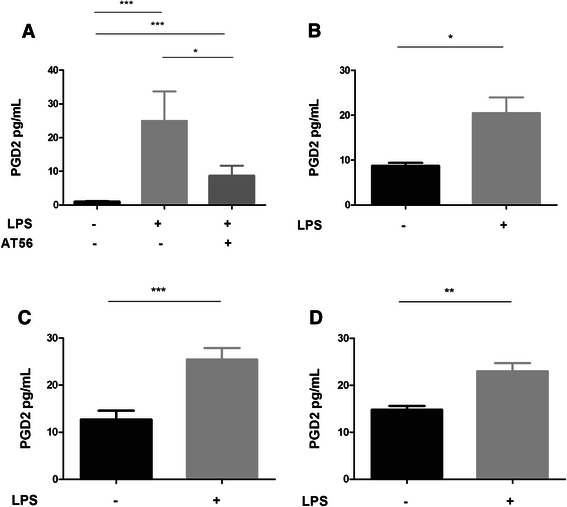
Enteric nervous system and PGD2 production in inflammatory conditions. Primary culture of ENS with and without LPS +/− AT-56 (95 μM) (**a**) (*n* = 12); coculture of ECG with neurons +/− LPS (**b**), EGCs culture +/− LPS (**c**), Neurons culture +/− LPS (**d**). Data were expressed as mean +/− SEM (*n* = 8), (Kruskall-Wallis test followed by Dunn’s *post hoc* test was used for Fig. 6a, Mann–Whitney test was used for Fig 6**b**, **c**, **d**)

First, L-PGDS expression by glial and neuronal cells in rat ENS primary culture was verified by immunostaining (Fig 6a-b). In contrast, HPGDS was not detected in rat ENS primary culture (data not provided). In order to evaluate the capacity of the ENS to produce PGD2 in proinflammatory conditions, primary cultures were treated for 24 h with LPS without or with AT-56, a specific competitive L-PGDS inhibitor. LPS was used to induce an inflammatory stress. We showed that 1/ the ENS produced PGD2 in response to LPS, and 2/ at the IC50 concentration (95 μM) AT-56 reduced PGD2 production in response to LPS (Fig 7a). These results confirmed that the ENS is able to produce PGD2 in proinflammatory conditions through L-PGDS. To determine the contribution of neurons and glial cells to the production of PGD2, a neuro-glial coculture model, in which neurons were plated on a glass coverslip suspended above a glia layer, was treated with LPS for 24 h. LPS treatment of the coculture model induced increased PGD2 production, as observed with ENS primary culture (Fig 7b). After 6 days’ co-culture, glial cells and neurons were then separated and treated with LPS for 24 h (Fig 7c-d). We observed increased PGD2 production in isolated neurons and glial cells, suggesting that both enteric neurons and EGCs were able to produce PGD2 in proinflammatory conditions.

## Discussion

Our work showed for the first time that there is activation of the entire PGD2 metabolic pathway by increase in mRNA expression of cPLA2-alpha, COX1, COX2 and L-PGDS in the inflamed zone of the colonic mucosa in active CD. Activation leads to increased PGD2 levels in inflamed colonic mucosa. Furthermore, our results showed that the ENS, both cellular components, ECG and neurons, can contribute to PGD2 production in response to proinflammatory stress.

In colonic biopsies of CD patients, we reported two activation profiles respectively associated with TNF-alpha and IL1B proinflammatory cytokines expression: a total activation profile involving TNF-alpha, IL1B, cPLA2-alpha, COX1, COX2 and L-PGDS, and intermediate activation profile, involving TNF-alpha, cPLA2-alpha and COX1 observed both in the normal zone of colonic mucosa in active and quiescent CD. This intermediate profile is found in the normal zone of colonic mucosa in the 15 patients with active CD but only in 5 patients with quiescent CD. Among quiescent patients, these five patients did not show any obvious clinical differences from other quiescent patients. These five patients presented a transcriptional status equivalent to that of active CD patients. No histological analysis was done on these colonic biopsy specimens. Such analysis could clarify this result which could be secondary to the accumulation of inflammatory cells in the tissue. On the other hand, to identify other actors implicated in the signaling pathways involved in these two distinct inflammatory profiles, it would be interesting to compare the transcriptome of patients with active and quiescent CD by performing microarray studies.

Interestingly, COX1 mRNA expression was upregulated in all patients with active CD and in five patients with quiescent CD. COX1 is usually known to be a constitutive enzyme playing physiological roles, not involved in inflammatory responses [30]. However, high expression of COX1 has already been reported in gastric mucosa in inflammatory situations, where COX1-activity was involved in thromboxane A2 (TXA2) synthesis [31]. Hawkey et al. have reported increased TXA2 synthesis in non-inflamed rectal mucosa of CD patients [32], suggesting that COX1-TXA2 activation, leading to platelet aggregability and local vasoconstriction, is also of importance in CD. Therefore, COX1 and COX2 are probably both involved in mucosal integrity through different lipid mediators.

In accordance with a previous study [33, 34], we showed up-regulation of IL1B and COX2 expression in the inflamed zone of the colon in patients with active CD. A significant increase in mucosal expression of COX2, the main enzyme responsible for PG production in inflamed mucosa had been already reported [33, 34]. Here, we showed, for the first time, an up-regulation of L-PGDS expression and an increased synthesis of PGD2 in CD. Our data are in keeping with recent results from Hokari *et al.* in UC [12]. They demonstrated that L-PGDS was up-regulated in severely inflamed mucosa and correlated with disease activity, suggesting inflammatory action. The mechanism by which the L-PGDS gene is up-regulated in inflammatory situations is partially described. Fujimory *et al*. demonstrated that L-PGDS gene expression is up-regulated by IL-1β [35]. In rats, two NF-Kappa B elements, present on the L-PGDS gene promoter region, are essential to up-regulation. Conversely, HPGDS expression was not increased in the inflamed mucosa of patients with active disease. In the gut, although HPGS is the most important form of PGDS, expressed by intestinal epithelial cells, mast cells and fibroblasts [7, 36], our data suggests that PGD2 derived from L-PGDS plays a greater role in the pathophysiology of CD.

In our study, we showed that PPARG is down regulated in inflamed colonic mucosa of CD patients with active disease. The colonic expression of the two distinct PGD2 receptors DP1 and DP2 in colonic biopsy specimens from patients with CD did not differ significantly from that in controls except for the expression of DP2 specifically reduced in inflamed colonic mucosa of CD patients with active disease compared with mucosa of CD patients with quiescent disease. The biological effect of PGD2 on intestinal inflammation remains widely debated. Only, few studies analyzed the role of PGD2 in an intestinal inflammatory process, with opposite roles [5, 6, 37, 38]. Functional duality of PGD2 is probably based on the differential expression in human IBD of the three potential receptors activated by PGD2 or its metabolite, 15dPGJ2. 15dPGJ2 plays an anti-inflammatory or immunomodulatory role through PPARG, which is expressed at high levels in the colonic epithelium. The downregulation of PPARG suggests the inability of 15dPGJ2, PGD2 metabolite, to execute completely its anti-inflammatory effects. The two distinct PGD2 receptors DP1 and DP2 are G protein coupled but signal via different second messengers. Engagement of DP1 results in protein kinase A activation [39] while DP2 acts to raise intracellular calcium levels [40]. PGD2 and 15dPGJ2 are both ligands for DP1 and DP2 [41]. Activation of DP1 leads to beneficial effects. Reduction of granulocyte infiltration through DP1 activation was observed in a colitis model [5]. In colon biopsies of patients with UC, Vong *et al*. reported that PGD2 contributes to maintaining remission in patients. They observed increased PGD2 synthesis and increased DP1 receptor expression [6]. Some studies also showed that DP1 receptor activation stimulates mucin secretion, MUC2 and MUC5AC [42, 43]. The main function of mucins is to protect the colonic mucosa [44]. MUC2 and MUC5AC are involved in epithelial repair in IBD by acting on cell differentiation and growth [45]. The beneficial role of PGD2 in CD may also be attributed to DP1 activation. PGD2 also plays an important role through DP2 activation by down-regulating neutrophil infiltration into the mucosa in acute colitis. In a sepsis mouse model, PGD2 and 15dPGJ2 were significantly increased in peritoneal fluid. Furthermore, COX2 and L-PGDS were up-regulated in the peritoneal exudate cells. In this model, DP2−/− mice exhibited a significantly higher accumulation of neutrophils into the peritoneal cavity compared with wild type mice [40].

Our study provides evidence on the origin of PGD2 in colitis. Enteric neurons and enteric glial cells are potent modulators of the intestinal epithelial barrier (IEB) and are involved in pathologies associated with altered barrier functions. EGC and neurons produce soluble factors known to regulate intestinal epithelial barrier functions [14, 21, 24, 46]. PGD2 also seems to play a role in this relationship. Previously, we reported that EGCs express L-PGDS and are a cellular source of 15dPGJ2, which regulate epithelial cell proliferation and differentiation *via* PPARG activation [23]. Here, we first demonstrated that enteric neurons express L-PGDS but not HPGDS. Secondly, we showed that neurons and glial cells secrete PGD2 through L-PGDS in proinflammatory conditions. Thus, the ENS is a source of PGD2 in response to inflammatory stress which regulates IEB functions.

These data are consistent with the ENS’s involvement in IBD pathogenesis. Altogether, enteric neurons, enteric glial cells and the IEB constitute a functional entity, the digestive neuronal-glial-epithelial unit, based on physical proximity and paracrin relations between its components [14]. The ENS is known to regulate the gut homeostasis process, and, in particular, intestinal epithelial barrier functions, *via* the release of various mediators [14]. In colitis, epithelial function is impaired, leading to elevated bacterial translocation. In the submucosal plexus, neurons and glial cells expressing Toll-like receptor 4, may participate in an adaptative reaction and may be engaged in the defense responses of the intestinal barrier [47]. This cellular reaction is similar to the proinflammatory process named reactive astrogliosis in the central nervous system (CNS). Immediately after CNS injury, glial cells undergo rapid morphological changes. Interestingly, L-PGDS, which is expressed by glial cells in the central nervous system, participates in reactive gliosis in an autocrine or paracrine manner, and may have pathological implications in neuroinflammatory diseases.

## Conclusion

Emerging data suggest that paracrine communication between enteric neurons, glial cells and IEC regulate key functions and is involved in controlling IEB homeostasis. The PGD2 metabolic pathway implicating L-PGDS appears to be activated in Crohn’s disease. This study demonstrates that L-PGDS, expressed in intestinal neurons and glial cells, is up-regulated in the inflamed colonic mucosa of patients with active CD. More detailed studies are necessary to elucidate the mechanism relating to involvement of the PGD2 pathway in CD. The next step will be identification of PGD2 targets in the IEB. It may reveal new targeted therapeutic options in CD.

## Abbreviations

CD: Crohn’s Disease
CNS: Central Nervous System
COX: Cyclooxygenases
cPLA2-alpha: Cytoplasmic Phospholipase A2
DP: D-type prostanoid
ECGs: Enteric Glial Cells
ENS: Enteric Nervous System
HPGDS: Hematopoietic Prostaglandin D Synthase
IBD: Inflammatory bowel diseases
IL1B: Interleukin 1, beta
L-PGDS: Lipocalin-type Prostaglandin D Synthase
LPS: Lipopolysaccharide
MRPS6: Mitochondrial ribosomal protein S6
PG: Prostaglandins
PGD2: Prostaglandin D2
PGH2: Prostaglandin H2
PGDS: Prostaglandin D Synthase
PLA2G4A: Phospholipase A2, groupe IVA
PPARG: Peroxysome Proliferator-Activated Receptor Gamma
PTGDR: Prostaglandin D2 Receptor
PTGDR2: Prostaglandin D2 Receptor 2
PTGDS: Prostaglandin D2 synthase
PTGS1: Prostaglandin-endoperoxide synthase 1
PTGS2: Prostaglandin-endoperoxide synthase 2
IEB: Intestinal Epithelial Barrier
qRT-PCR: Quantitative Reverse Polymerase Chain Reaction
TNF-alpha: Tumor Necrosis Factor-alpha
TXA2: Thromboxane A2
UC: Ulcerative Colitis
